# Climate structuring of *Batrachochytrium dendrobatidis* infection in the threatened amphibians of the northern Western Ghats, India

**DOI:** 10.1098/rsos.180211

**Published:** 2018-06-13

**Authors:** Christopher J. Thorpe, Todd R. Lewis, Matthew C. Fisher, Claudia J. Wierzbicki, Siddharth Kulkarni, David Pryce, Lewis Davies, Aparna Watve, Mairi E. Knight

**Affiliations:** 1 Ecology, Behaviour and Evolution Research Group, School of Biological and Marine Sciences, University of Plymouth, Drake Circus, Plymouth, Devon PL4 8AA, UK; 2 Westfield, 4 Worgret Road, Wareham, Dorset BH20 4PJ, UK; 3 Department of Infectious Disease Epidemiology, Imperial College London, London W2 1PG, UK; 4 Department of Biological Sciences, George Washington University, 2121 I St NW, Washington, DC 20052, USA; 5 Tata Institute of Social Sciences, Apsinga Road, PO Box No. 09, Tuljapur 413 601, District-Osmanabad, Maharashtra, India

**Keywords:** Western Ghats, chytrid, amphibians, caecilians, plateaus

## Abstract

*Batrachochytrium dendrobatidis* (*Bd*) is a pathogen killing amphibians worldwide. Its impact across much of Asia is poorly characterized. This study systematically surveyed amphibians for *Bd* across rocky plateaus in the northern section of the Western Ghats biodiversity hotspot, India, including the first surveys of the plateaus in the coastal region. These ecosystems offer an epidemiological model system since they are characterized by differing levels of connectivity, edaphic and climatic conditions, and anthropogenic stressors. One hundred and eighteen individuals of 21 species of Anura and Apoda on 13 plateaus ranging from 67 to 1179 m above sea level and 15.89 to 17.92° North latitude were sampled. Using qPCR protocols, 79% of species and 27% of individuals tested were positive for *Bd*. This is the first record of *Bd* in caecilians in India, the Critically Endangered *Xanthophryne tigerina* and Endangered *Fejervarya cf. sahyadris*. Mean site prevalence was 28.15%. Prevalence below the escarpment was 31.2% and 25.4% above. The intensity of infection (GE) showed the reverse pattern. Infection may be related to elevational temperature changes, thermal exclusion, inter-site connectivity and anthropogenic disturbance. Coastal plateaus may be thermal refuges from *Bd*. Infected amphibians represented a wide range of ecological traits posing interesting questions about transmission routes.

## 1. Introduction

*Batrachochytrium dendrobatidis* (*Bd*) [1] is an aggressive species of chytrid fungus that can cause the lethal amphibian infection chytridiomycosis [2]. Following the emergence of a hypervirulent lineage of *Bd*, the global panzootic lineage (*Bd*GPL) [3], in the early twentieth century, the pathogen has been responsible for the loss of entire species [4] and is considered a significant threat wherever it is found [5]. The presence of *Bd* in the Western Ghats (WG) biodiversity hotspot [6] has been known since 2011 [7], with its known range in the WG extended in 2015 [8], and chytridiomycosis was reported from the northern WG in 2013 [9]. It was identified as an endemic Asian strain in 2013 [9]. It remains unclear what factors regulate the distribution of *Bd* in the WG and its transmission, and what the reasons are for its current generally sub-lethal state in the region. In a peculiar twist of fate, regions that are home to the world's greatest amphibian diversity are also most suitable for *Bd* [10]. In a global model, Olson *et al*. [2] found the entire WG to be suitable habitat for the pathogen [2].

The WG in southwest India occupy just 5% of the country's land mass and yet are home to some 42% of its amphibian species (approx. 161 species) [11,12]. Not only are the WG highly specious but many of its amphibian species are rare, with 87% being WG endemics [12]. The amphibians that are endemic to the rocky plateaus (plateaus) face both proximate and ultimate threats including climate change [13], and regional stressors (population growth [14]), along with rapid habitat loss through mining, tourism and wind turbine installations [15–18]. They also face challenges from pathogens such as the fungus *Batrachochytrium dendrobatidis* (*Bd*) [7–9]. Systematic studies on *Bd* in Asia are under-represented in the literature [19], and this study aims to help to address that shortfall.

The three studies that have been published so far examining *Bd* infection in the Western Ghats biodiversity hotspot cover almost the entire length of the WG and report widely differing levels of infection ranging from 0.6% [7] to 25% [9]. The most geographically extensive study, covering the northern, central and southern WG, reported an infection rate of 1.6% [8]. All three studies excluded the low-lying sites between the coast and the hills. Molur *et al.* [8] published a predictive model showing higher risk of infection south of approximately 14.5°N in the central section of the WG.

This present study adds considerably to the previous WG publications by surveying low elevation sites for the first time. In addition, this is the first study in the WG to offer data on habitat specific infection rates, and infection patterns across a wide range of elevations. Such data are highly important as the high-level plateaus are becoming recognized as centres of endemism for a number of taxonomic groups and data on all threats are urgently needed for their effective management [17–20].

## 2. Methods

### 2.1. Study area

**Figure 1.**
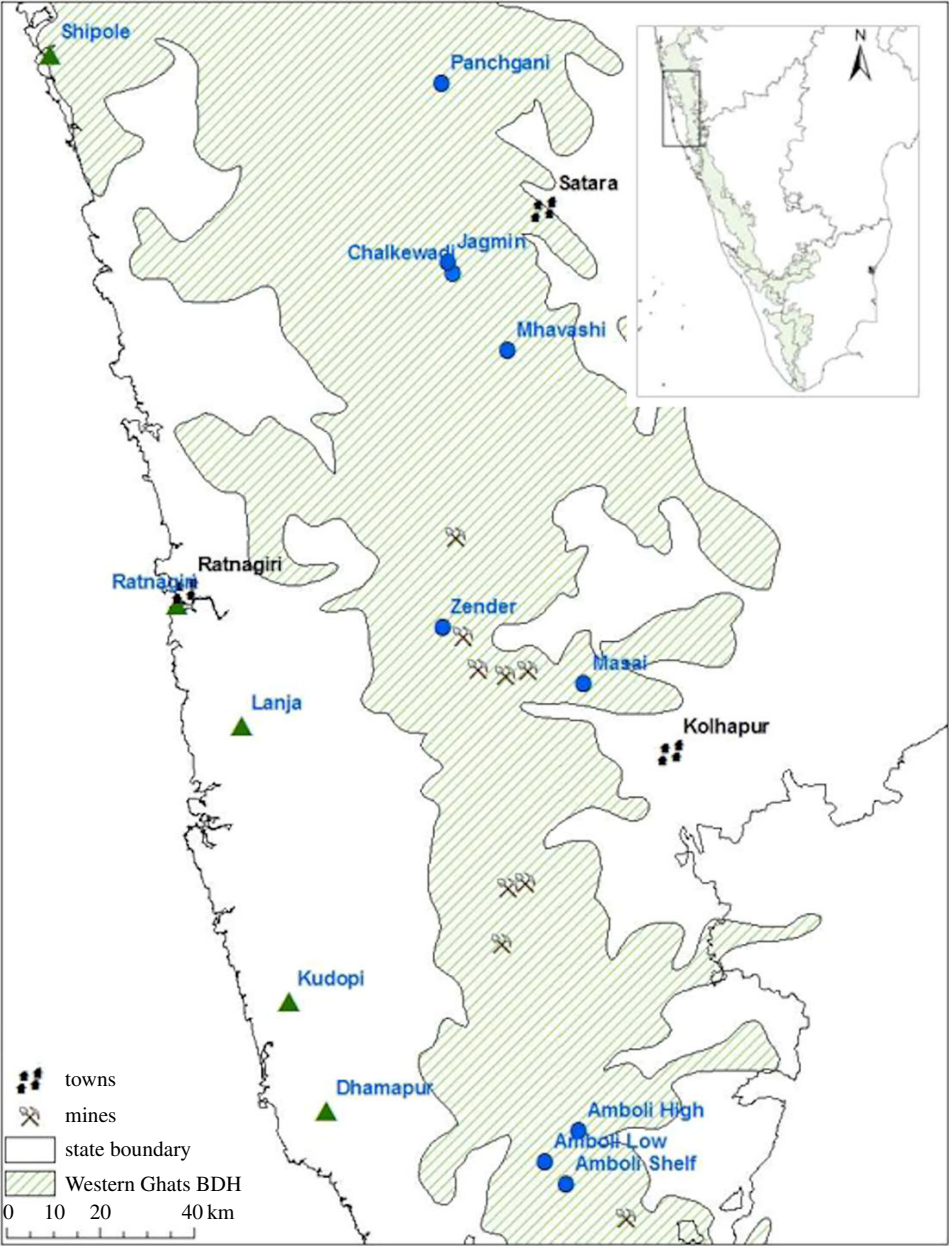
Map of study area, with study area location within India (inset). Nearest large towns are shown for reference points. Blue circles denote plateaus in the High Region and green triangles those in the Low Region. Mine sites are indicated to reflect one of the risks to these sites. The biodiversity hotspot (BDH) [6] outline was created in ArcGIS based on data downloaded from ESRI (Environmental Systems Research Institute, Redlands, California, USA).

The WG are a chain of hills some 1500 km in length running parallel and slightly inland from the south west coast of India from the Maharashtra/Gujarat state border to the country's southern tip (figure 1). They are part of the Western Ghats--Sri Lanka biodiversity hotspot [6] and the eighth ‘hottest’ hotspot on the planet [21]. Unlike the granitic central and southern sections, the northern section in western Maharashtra, known as the Deccan Traps (DT), is formed from basalt. The plateaus are ferricretes of laterite forming hilltop carapaces above the escarpment on the western edge of the WG rising to 1200 m above sea level (m), with extensive low-lying plateaus below in an area known as the Konkan [22]. Temperatures range from 15°–40°C in the Konkan and 4°–42°C above the escarpment [23]. The higher elevation amphibian populations are exposed to lower temperatures that may be more conducive to *Bd* infection [24].

### 2.2. Amphibian sampling

Amphibians were sampled from 13 representative plateaus situated in the northern WG both above and below the North--South escarpment in western Maharashtra during the early monsoon in 2013 and 2014 (late July–early August; figure 1, electronic supplementary material, table S3). Plateaus were selected to represent the latitudinal and elevation extent of laterite in western Maharashtra together with the range of land uses in each region (electronic supplementary material, table S3). Visual encounter surveys with supplementary refugia searching were performed along four 6 m by 100 m transects on each plateau in each year [25,26]. Anthropogenic disturbance factors assessed at each site were: removal of loose rocks, surfaced road, unsurfaced road, built structures on the plateau, domesticated animal grazing, surfaced road within 200 m of plateau, tourism, part conversion to plantation, adjacent built structures and importation of topsoil. Sites with 0–3 factors were considered to have low levels of disturbance, those with 4–7 medium disturbance and with 8+ high disturbance (electronic supplementary material, table S3).

Sampled amphibians were identified by morphological comparison with the best literature available (including [27–45]). Many WG amphibians are taxonomically cryptic or unstable [43–45]; in cases where species-level identification may be in doubt we use ‘cf.’.

### 2.3. Field sampling and laboratory techniques

Amphibians were all hand-captured, individually bagged and transported to shelter where skin swabs were taken from ventral surfaces. Swabbing was performed by a pair of surveyors using sterile cotton tip swabs (T/S16-B; Technical Service Consultants Ltd) [46,47]. The ventrum, drink patch, thighs and toe-webbing of each adult anuran and metamorph were swabbed multiple times following published standardized protocols [48]. For caecilians a simpler approach of multiple swab strokes along the whole of the body was used. Swabs were broken off into sterile vials of 99% ethanol. Disposable equipment (latex gloves and polythene bags) were replenished between specimens and sites. Other equipment was sterilized using VirkonS™ solution or dried to minimize cross-contamination.

DNA extraction from the swabs followed the protocol described by Boyle *et al*. [46,49]. A quantitative real-time polymerase chain reaction (qPCR) diagnostic assay was used with *Bd* specific probes for the ribosomal gene region ITS-1/5.8S [49]. The qPCR was run on an Applied Biosystems QuantStudio 7 Flex Real-Time PCR System with an additional 10 cycles being added to the Boyle protocol (60 cycles total). Standards of known concentration of *Bd* DNA (100, 10, 1, 0.1 *Bd* GE (zoospore genomic equivalents)) were used as positive controls and standards together with no template control (NTC) of molecular grade water as a negative control. The samples were run in duplicate with single positives repeated. A positive result was a sample with a GE greater than 0.1 in both samples in a qPCR pair, a single was a sample for which only one of each qPCR pair had a GE greater than 0.1 and a weak signal was where only one out of four qPCR scores was greater than 0.1.

### 2.4. Environmental correlates

*Bd* is sensitive to a range of changes in the abiotic environment. Plateau soils are acidic, ranging as low as pH 4.9 which is below the *Bd* optimum of pH 6–8 [16,23,50]. In addition, *Bd* is temperature-sensitive, growing best between 17 and 25°C with an optimum of 23°C [50,51]. Temperature may also regulate the pathogenicity of *Bd*, with frogs exposed between 17 and 23°C more likely to die than those exposed at 27°C [52]. The fungus is reported to die when the temperature exceeds 30°C, a level exceeded at times on all plateaus [10,16]. Lower seasonal temperatures, such as those during the monsoon at higher elevations in the WG, are known to favour the pathogen [50]. The plateaus are mosaics of microhabitats set in a heterogeneous landscape with unknown degrees of connectivity for *Bd* [15,16,23,53]. Microhabitats include expanses of exposed rock often with associated loose rocks. Exposed rock absorbs solar radiation giving it a surface temperature higher than the air, perhaps creating microclimates that may reduce the intensity and presence of the pathogen [54]. Other microhabitats include loose rocks and fissures offering refugia from dry areas and excessive temperatures [55–57]. Optimum rainfall for *Bd* is reported to be between 1500 and 2500 mm a year. Only the low lying plateaus fall within this range, the plateaus along the top of the escarpment receiving between 4000 and 9000 mm, with the exception of Masai which is east of the ridge and may be drier [10,55].

Macro-environment and physiochemical data were recorded for each site: air, soil and water temperature (°C) and pool pH using a calibrated electrical probe (Hanna Instruments™ HI 9064); elevation (m), latitude and longitude for the start and end of each transect using a hand held GPS (Garmin™ 60csx GPS).

## 3. Permission for fieldwork

Permission for accessing biodiversity in India including the fieldwork was granted by the National Biodiversity Authority, India, to C. J. Thorpe, permit number MC200621. The permit authorizes some other authors to assist with sampling.

## 4. Data analysis

To help make analytical comparisons at a range of spatial scales, the study area was divided into two regions: above the Western Ghats escarpment (High Region), and below it (Low Region). The dividing line was set at 700 m above sea level. Each region was arbitrarily sub-divided into three latitudinal groups North, Central and South (electronic supplementary material, table S3). Correlations between site elevation, temperature and pH were explored through Pearson product moment correlation for parametric data and Spearman rank correlation for non-parametric data. To assess the impact of the spatial arrangement and disturbance on *Bd* GE values, general linear model (GLM) and one-way ANOVA analyses were performed to investigate elevation, latitude, region, disturbance type and disturbance intensity. Results are reported with a confidence level (CL) of 95% together with upper and lower bi-nomial confidence bounds (CB), which are the outer values of the confidence interval that are expressed as percentages [58,59].

## 5. Results

### 5.1. Overview of *Bd* infection in the study area

A total of 118 sample swabs were taken from individuals belonging to 2 orders, 6 families, 14 genera and 19 taxa (table 1; electronic supplementary material, tables S4 and S5). Seventy-nine per cent of the taxa tested had individuals positive for *Bd* (table 1; electronic supplementary material, tables S4 and S5). The study does not provide an inventory of infection for the area as it only covered one ecosystem and complete detection of both species and infection is problematic [60]. Total prevalence in the sample was 11% (95% CL; CB 7–19), 22% (95% CL; CB 15–31) if single positives were included and 27% (95% CL; CB 19–36) including weak positives. As in other WG studies, all GE values recovered were low and all those with a single GE value greater than 0.1 were included in the analysis (table 1; electronic supplementary material, tables S4 and S5 [7–9,61]).

**Table 1.** Taxonomy, risk and infection as prevalence. Where nomenclature is uncertain the rules of the International Code of Zoological Nomenclature (ICZN) have been followed. Where identification is hampered by cryptic species a most likely identity is shown with the prefix ‘cf’. IUCN threat status and known habitat associations accessed 10/02/2017 [51]. NA, not assessed; DD, Data Deficient; LC, Least Concern; EN, Endangered; CR, Critically Endangered. Prevalence is the percentage of the sample tested positive for *Bd.*

order	family	taxa	IUCN	*N*	prevalence (%)	CB (%)
Anura	Bufonidae	*Duttaphrynus melanostictus*	LC	7	43	10–82
Anura	Bufonidae	*Xanthophryne tigerina*	CR	15	6.7	1–32
Anura	Dicroglossidae	*Euphlyctis* cf. *cyanophlyctis*	LC	2	0	0
Anura	Dicroglossidae	*Fejervarya* cf. *brevipalmata*	DD	9	11	0.003–0.48
Anura	Dicroglossidae	*Fejervarya* cf. *caperata*	DD	4	75	19–99
Anura	Dicroglossidae	*Fejervarya* cf. *cepfi*	NA	7	14	0.01–58
Anura	Dicroglossidae	*Fejervarya* cf. *sahyadris*	EN	14	36	13–65
Anura	Dicroglossidae	*Fejervarya* sp.		10	33	7–65
Anura	Dicroglossidae	*Hoplobatrachus tigerinus*	LC	9	56	21–86
Anura	Dicroglossidae	*Sphaerotheca dobsonii*	LC	5	20	1–72
Anura	Micorhylidae	*Microhyla ornata*	LC	1	0	0
Anura	Micorhylidae	*Uperodon globulosus*	LC	1	0	0
Anura	Ranixalidae	*Indirana* cf. *chiravesi*	LC	3	33	1–91
Anura	Rhacophoridae	*Pseudophilautus* sp.		1	0	0
Anura	Rhacophoridae	*Raorchestes* g*hatei*	NA	5	60	15–95
Gymnophiona	Indotyphlidae	*Gegeneophis* cf*. ramaswamii*	LC	5	20	1–72
Gymnophiona	Indotyphlidae	*Gegeneophis seshachari*	DD	9	44	14–79
Gymnophiona	Indotyphlidae	*Indotyphlus* cf. *battersbyi*	DD	2	50	1–99
Gymnophiona	Indotyphlidae	*Indotyphlus maharashtraensis*	DD	3	33	1–91

All four species of caecilian found in the study were infected (table 1; electronic supplementary material, tables S4 and S5). The critically endangered Amboli toad, *Xanthophryne tigerina,* was infected with low prevalence (6.7%; 95% CL; CB 1–32) as were 33% of the endangered frog *Fejervarya* cf. *sahyadris* in the sample (95% CL; CB 13–65) [51]. One site, Amboli High, returned no positives out of six *Xanthophryne tigerina* samples. Amboli High was the only site without any positive results. No amphibians were detected with external signs of chytridiomycosis.

### 5.2. Spatial distribution of *Bd* infection

**Figure 2:**
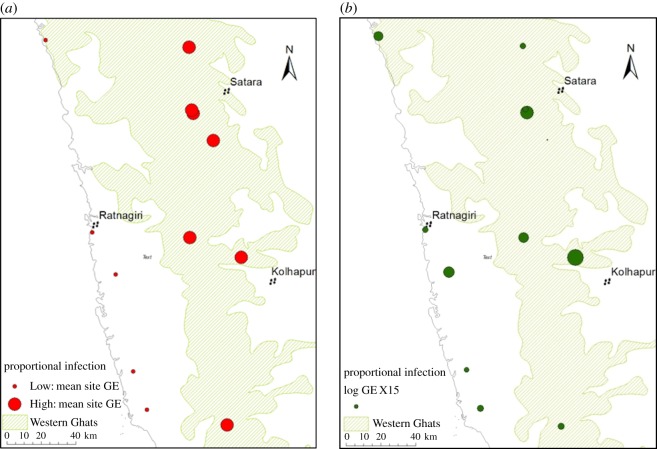
(*a*) The proportional difference in regional values of mean site GE values. (*b*) The individual site mean GE.

Site prevalence varied between 0 and 50% and species infection rate was 0–60% (table 1; electronic supplementary material, table S4). Macroscale variations in GE values were found with the regions exhibiting a significant 10-fold difference in mean GE (ANOVA, *F*_1,2_ = 3.99, *p* = 0.06) (electronic supplementary material, table S2; figure 2*a*,*b*). The Low Region had more infected individuals (56%; 95% CL; CB 26–62) than the High Region (44%; 95% CL; CB 37–56). Individual GE value increased with elevation (ANOVA, *F*_11,20_ = 4.85, *p* < 0.01).

### 5.3. Environmental relationships with *Bd* infection

As both water temperature and pH covaried with elevation, and pH with water temperature, elevation alone was used to explore spatial relationships (electronic supplementary material, table S1). Temperature in the Low Region had a notable maximum of 36.4°C (mean 30.9°C), much higher than above the escarpment at 28.3°C (mean 22.5°C; minimum 19.3°C) (table 2). Both regions had minimum pH values below the *Bd* optimum although mean values were within the pathogen's tolerance range (table 2).

**Table 2.** Physio-chemical parameters described for the survey area as a whole and the two regions. Temperature = water temperature in °C.

variable	Region	mean	minimum	maximum
temperature	Low	30.9	26.2	36.4
temperature	High	22.5	19.3	28.3
temperature	all	24.8	19.3	36.4
pH	Low	6.7	5.0	9.6
pH	High	7.6	5.3	12.2
pH	all	7.3	5.0	12.2

**Figure 3:**
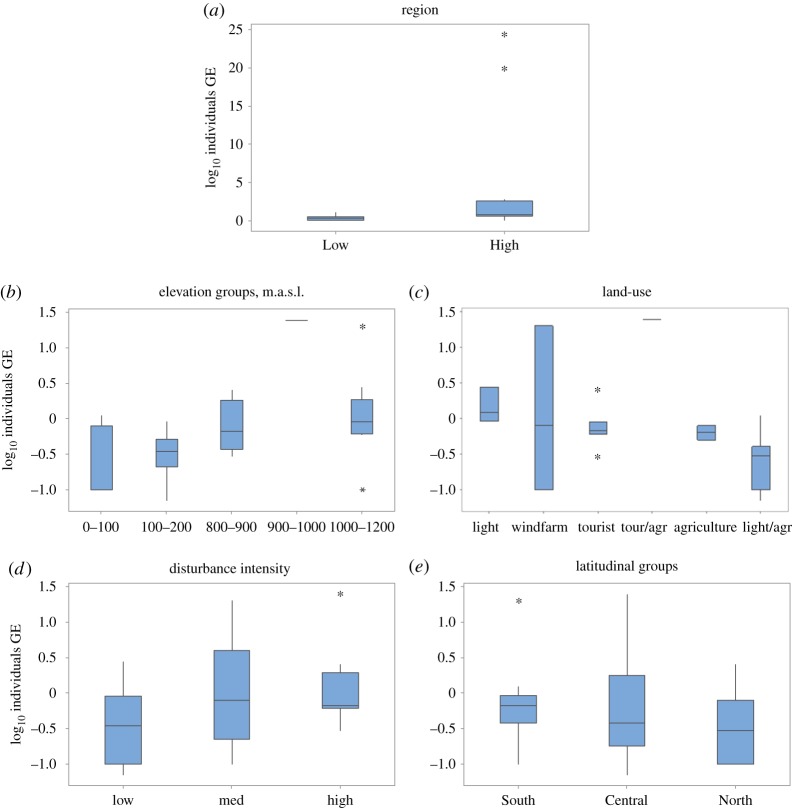
Log_10_ transformed GE data for individuals. Quartile 2 and 3 are shaded with the dividing line as the median. The whiskers indicate quartiles 1 and 4. Outliers are indicated by asterisks. (*a*) Regions (above, High, and below, Low, the Western Ghats escarpment). GLM ANOVA results for the other classes: (*b*) elevation groups, *F* = 12.77, d.f._factor_ = 1, d.f._error_ = 24, *p* < 0.01; (*c*) land-use, *F* = 10.88, d.f._factor_ = 1, d.f._error_ = 24, *p* < 0.01; (*d*) disturbance intensity, *F* = 2.99, d.f._factor_ = 4, d.f._error_ = 24, *p* < 0.05; (*e*) latitudinal groups, *F* = 3.33, d.f._factor_ = 1, d.f._error_ = 24, *p* = 0.08.

GLM ANOVA analysis was used to assess the factors regulating the distribution of infection intensity (figure 3*a–e*). Plateau elevation had the greatest impact showing an upward trend with elevation (figure 3*b*). Land-use was the second most crucial factor: agriculture had a negative impact on sites below the escarpment, and above the escarpment the type of land-use did not have a clear impact (figure 3*c*). The intensity of disturbance was related to an increasing trend in infection intensity (figure 3*d*). Latitude, which includes Low and High Region sites in each class, suggests a limited decreasing trend with increasing latitude (figure 3*e*).

## 6. Discussion

There was widespread but low intensity infection of *Bd* on almost all the plateaus sampled except for one, Amboli High, and in 79% of the amphibian species examined on the rocky plateaus in the northern WG (table 1; electronic supplementary material, tables S3 and S4). These are the first records of infection in the critically endangered Amboli toad, *Xanthophryne tigerina;* the endangered frog *Fejervarya* cf. *sahyadris;* and four species of caecilian, *Gegeneophis* cf*. ramaswamii, Gegeneophis seshachari, Indotyphlus* cf. *battersbyi* and *Indotyphlus maharashtraensis* [62]. Three of the caecilian species are described by the IUCN as Data Deficient (table 1). Infection was detected in two species yet to be assessed by the IUCN, *Fejervarya* cf. *cepfi* and *Raorchestes* cf. *ghatei*; and two other Data Deficient species, *Fejervarya* cf. *brevipalmata* and *Fejervarya* cf. *caperata* (table 1). This is the first study to investigate *Bd* infection in low lying coastal sites, where there was higher prevalence but lower intensity infection than on sites above the WG escarpment (electronic supplementary material, tables S2 and S3; figure 3*a*). Site elevation, with its covariables, and disturbance intensity were the most significant explanatory factors in the pattern of *Bd* distribution (electronic supplementary material, tables S2 and S3; figure 3*b*,*c*). However, another explanation, not explored here, for the observed pattern in *Bd* distribution is that the amphibian populations are relics of ancient dispersals isolated from the pathogen's transmission vectors [63].

### 6.1. Low elevation plateaus are less conducive to *Bd* but with greater connectivity

Puschendorf *et al*. [54] suggest that disease-free amphibian refuges are created in drier areas with temperatures above those tolerated by *Bd* [54]. We suggest our findings, with lower infection intensities on the Konkan plateaus, support them as a possible refugia for some amphibian species from *Bd*. However, the higher prevalence on plateaus below the escarpment is more difficult to explain. The plateaus' specific environment derived from their open habitat may have created thermal refugia from *Bd* in rock pools and surrounding habitats where temperatures exceed the pathogen's upper thermal tolerance, but it should also restrict transmission. The pools are scattered across plateaus with surface temperatures, especially on the exposed rock, greatly more than the pathogen's thermal maximum, which should restrict the pathogen's persistence and transmission [23,64].

Low Region water temperatures were higher than those above the escarpment, on average 30.9°C, with a maximum of 36.4°C, a figure well above published critical zoospore thermal thresholds of 23–28°C (tables 1 and 2; figure 3*a*; electronic supplementary material, table S2 and S3) [2,10,50,65]. Even more lethal are the rock surface temperatures which can exceed 50°C [23]. The region is also drier, with less rain and fewer wet days than above the escarpment. The annual rainfall falls within the pathogen's preferred rainfall range of 1500–2500 mm but it only rains for five months a year, with the remaining seven months being almost completely dry with very low relative humidity [10,23,65,66]. Infection intensity decreased slightly with increasing latitude possibly reflecting the latitudinal decline in the number of wet days (figure 3*e*) [56,57,66].

Regional differences in habitat and micro-habitat availability may also help explain the pattern in *Bd* distribution, through behavioural mitigation where amphibians move to, or persist on, plateaus with micro-habitats that are refugia from *Bd* [55,57,67]. Conversely, stream micro-habitats offer one possible transmission route in the Low Region, where streams are more frequent [55]. Sub-tropical stream-breeders are more susceptible to *Bd* and may be disease vectors with the pathogen spreading from streams into the terrestrial realm [67,68]. Increased landscape connectivity in the Low Region, resulting from lower inter-site variation in elevation (Low Region 103 m, High Region 370 m), may enable greater inter-site transmission through amphibians dispersing between plateaus, explaining the higher prevalence and supporting the findings of Heard *et al.* [69] (electronic supplementary material, table S2 and S3) [53,70]. The picture is complex though as some refugia such as woody plants and large loose rocks may enable behavioural avoidance of excessive temperatures for both amphibians and *Bd* [51,69,71]. This idea possibly supported by the results for the four species of caecilian in the study which have similar prevalence to non-fossorial taxa (table 1; electronic supplementary material, tables S4 and S5). They are frequently found under loose rocks where temperatures are tolerable for *Bd* and are close to streams which may be used by stream-breeding anuran vectors [55,67].

### 6.2. *Bd* in the High Region

Despite the High Region offering a more equitable temperature range for *Bd* with a mean of 22.5°C, within the pathogen's *in vitro* optimum of 17–25°C, prevalence was less than below the escarpment where the pathogen's upper limit was often exceeded. This suggests the High Region's greater topographical heterogeneity produces barriers to transmission which may explain the lower number of infected individuals (tables 1 and 2; electronic supplementary material, tables S2–S4; [10,50,51]).

Individual GE loadings were greater above the escarpment reflecting the High Region's optimal temperature (table 2 and electronic supplementary material, table S2; [10,51]). While excessive temperatures below the escarpment may regulate the pathogen through mortality the High Region's lower temperature regime may encourage *Bd,* even when the temperature falls below the organism's lower optimum value (17°C). A temperature of 4°C was used by Voyles *et al*. [57] as their minimum in a study assessing the impact of temperature regimes on *Bd* life history; the same minimum temperature was also recorded by Watve [23] on High Region plateaus. Their lower temperature regime resulted in extended zoospore longevity meaning zoospore numbers in water bodies could be expected to be greater than in warmer pools. The trait may lead to greater encounter rates and thus infection prevalence contrary to our findings [57]. However, they also found their low temperature regime increased zoospore production, which offers a plausible explanation for the elevated site GE values in the High Region.

### 6.3. The impact of anthropogenic disturbance on *Bd* distribution

Elevated prevalence close to human settlement is to be expected but with unknown causes [72]. The study found that Low Region plateaus, which are less isolated from human settlement, had higher prevalence than their more isolated High Region counterparts [72].

In addition to proximity of human habitation, changes in land-use influences amphibian distribution, and possibly their susceptibility and exposure to disease [55,73,74]. Sites near human habitation are likely to have anthropogenic land-uses. We found land-uses differed either side of the escarpment. Land-use had an impact on mean individual infection intensity with Low Region agricultural sites having higher infection intensity than the nearest sites with limited disturbance (figure 3*c*). Land-use in the High Region had a negligible impact on mean individual infection levels (figure 3*c*). Sites with little disturbance, on our arbitrary scale, had lower mean individual GE compared to plateaus with higher disturbance (figure 3*d*). The actual mechanisms remain unclear, but we can support anthropogenic disturbance as a negative factor in *Bd* infection. The sites disturbed by tourism had amphibian assemblages dominated by generalist species, but this had no impact on mean individual infection intensity suggesting mobile species may not be pathogen vectors [55].

The impact of disturbance on *Bd* infection intensity was less than that of elevation (figure 3*b–d*). Site prevalence did not reflect land-use (electronic supplementary material, table S3; figure 3*b–e*). As all the sites with tourism were above the escarpment and elevation had the greater explanatory power, we believe spatially driven climate has more effect than land-use. There is a clearer indication that agriculture negatively impacts infection intensity as seen in our Low Region sites, where there is little inter-site variation in elevation but a significant difference in infection intensity (figure 3*c*).

## 7. Conclusion

It is clear that the *Bd* pathogen is very widely distributed in this area and anthropogenic land-use increases the infection risk. The infected plateau amphibians include several threatened and poorly understood species whose infection we record for the first time. None of the individuals that tested positive for *Bd* showed any external signs of chytridiomycosis. The disease has been reported in *Nyctibatrachus humayuni* from sites close to the northern edge of this survey [9]. The infection level reported here is well below the mortality threshold of 1–10 000 zoospores [75] and is more indicative of an historical infection, or species that are carriers that do not go on to develop chytridiomycosis [76–78]. The triggers for this low intensity infection to develop into a lethal outbreak of chytridiomycosis are unknown.

Transmission vectors are poorly understood globally as well as in the WG, but we would support the possible explanation of water birds as vectors with lapwing species (*Vanellus indicus*) being frequently observed on all the plateaus [79]. Proximity to human habitation is a risk factor but the mechanisms of transmission are unknown.

Until there is a better understanding of the mechanisms triggering benign *Bd* infection to become lethal chytridiomycosis, its presence should be considered in all future conservation policy decisions. Preservation of dispersed populations on sites with refugial properties, good connectivity and preservation of refugia on individual plateaus is essential in offering the best prospect of long-term species persistence [69,80]. The need for further work on modelling infection on a wider scale, especially in the low lying coastal areas, characterized here for the first time for *Bd*, is a priority. A study into the evolutionary history of *Bd* in the entire WG area would also help with its management. There is an urgency to determine the routes of transmission and triggers for the pathogen to become lethal. That urgency is illustrated by the 2015 publication of the addition of *Duttaphrynus melanostictus* to *Hoplobatrachus tigerinus* as invasive species in another biodiversity hotspot, Madagascar, [81]. Both of these species had high *Bd* prevalence in this study, 43% and 56% respectively. Resolution of these questions may be helped by a better understanding of the historical lineage of the *Bd* strain in the WG.

## Supplementary Material

S1 Supplementary material for: Thorpe et al. 2018. Climate structuring of Batrachochytrium dendrobatidis infection in the threatened amphibians of the northern Western Ghats, India Journal manuscript number: RSOS-170810

## Data accessibility

The raw data of infection intensity with species and site localities are available in the electronic supplementary material.
