# Supplementary material for: Climate structuring of Batrachochytrium dendrobatidis infection in the threatened amphibians of the northern Western Ghats, India
Source: R Soc Open Sci. 2018 Jun 13;5(6):180211. doi: 10.1098/rsos.180211 (PMC6030269; doi:10.1098/rsos.180211)
Supplement: S1 Supplementary material for: Thorpe et al. 2018. Climate structuring of Batrachochytrium dendrobatidis infection in the threatened amphibians of the northern Western Ghats, India Journal manuscript number: RSOS-170810 [file rsos180211supp1.docx]

S1. Supplementary material for:

**Thorpe et al. 2018. Climate structuring of *Batrachochytrium dendrobatidis* infection in the threatened amphibians of the northern Western Ghats, India.**

**Table 1.** Linear regression of physiochemical parameters. Temperature = Water Temperature; Elevation is site elevation above sea level.

| **Variable** | **Related to** | **R^2^** | **F** | **P** |
| --- | --- | --- | --- | --- |
| Temperature | Elevation | 0.71 | F_1,158_ =384.2 | < 0.001 |
| pH | Elevation | 0.095 | F_1,158_ =16.47 | < 0.001 |
| pH | Temperature | 0.214 | F_1,158_ = 42.75 | <0.001 |

**Table 2.** Distribution of mean positive GE values for individuals by geographical region. Standard Deviation-SD.

| **Region** | **Sample n** | **Mean GE** | **SD** | **Minimum** | **Maximum** |
| --- | --- | --- | --- | --- | --- |
| All Sites | 32 | 2.0 | 2.0 | 0.1 | 24.3 |
| High Region | 14 | 4.0 | 7.7 | 0.1 | 24.3 |
| Low Region | 18 | 0.4 | 0.3 | 0.1 | 1.1 |

**Table 3.** Site descriptions with prevalence and infection intensity. Land Use = disturbance types as the dominant form on a plateau; Lat. Group = latitudinal group; Elev = elevation in m above sea level, Dist. Int. = Disturbance Intensity and follows the additive formulae of factors in Table 1. Prev. = Prevalence is the percentage of the sample tested positive for *Bd.* GE = Genomic Equivalent; SD = Standard Deviation; Site GE = Mean Site Genomic Equivalent. Confidence Bounds as a % (CB) are quoted for a Confidence Level of 95%.

| **Site** | **Land Use** | **Region** | **Lat. Group** | **Elev.** | **Dist. Int.** | **n** | **Prev.** | **CB** | **Site GE** | **SD** | **Min.** | **Max.** |
| --- | --- | --- | --- | --- | --- | --- | --- | --- | --- | --- | --- | --- |
| Chalkewadi | Wind Turbines | High | North | 1157 | Medium | 8 | 25 | 3-65 | 10.3 | 13.44 | 0.8 | 19.8 |
| Jagmin | Light | High | North | 1179 | Low | 8 | 25 | 3-65 | 1.1 | 0.2 | 0.9 | 1.2 |
| Mhavashi | Wind Turbines | High | North | 1090 | Medium | 3 | 33 | 1-91 | 0.1 | * | 0.1 | 0.1 |
| Panchgani | Tourism | High | North | 1131 | High | 10 | 40 | 12-74 | 0.7 | 0.2 | 0.6 | 0.9 |
| Shipole | Light/ Agriculture | Low | North | 170 | Low | 11 | 46 | 17-77 | 0.5 | 0.3 | 0.3 | 0.9 |
| Masai | Tourism/  Agriculture | High | Central | 974 | High | 4 | 25 | 1-80 | 24.3 | * | 24.3 | 24.3 |
| Zenda | Light | High | Central | 1015 | Low | 4 | 50 | 7-93 | 2.8 | * | 2.8 | 2.8 |
| Ratnagiri | Agriculture | Low | Central | 67 | Medium | 5 | 20 | 1-72 | 0.5 | * | 0.5 | 0.5 |
| Lanja | Light Agriculture | Low | Central | 156 | Low | 13 | 38 | 14-68 | 0.3 | 0.1 | 0.1 | 0.4 |
| Amboli Low | Tourism | High | South | 809 | High | 16 | 25 | 7-52 | 1 | 1 | 0.3 | 0.9 |
| Amboli High | Light | High | South | 854 | Low | 6 | 0 | 0 | 0 | 0 | 0 | 0 |
| Kudopi | Light Agriculture | Low | South | 90 | Low | 16 | 39 | 15-65 | 0.3 | 0.4 | 0.1 | 1.1 |
| Dhamapur | Agriculture | Low | South | 85 | Medium | 8 | 13 | 9-19 | 0.8 | * | 0.8 | 0.8 |

**Table 4.** Positive results for *Bd* infection with species and plateau location. Mean GE is the mean of all qPCR where results >0.1. SD – indicates the Standard Deviation for the positive qPCR results reported as Mean GE, an * indicates weak results, a single replicate qPCR result.

| **Taxa** | **Site** | **Mean GE** | **SD** |
| --- | --- | --- | --- |
| *Fejervarya* sp. | Amboli Low | 2.53 | 2.30 |
| *Fejervarya* cf*. caperata* | Amboli Low | 0.67 | 0.64 |
| *Gegeneophis* cf. *ramaswamii* | Amboli Low | 0.67 | 0.57 |
| *Xanthophryne tigerina* | Amboli Low | 0.30 | * |
| *Duttaphrynus melanostictus* | Chalkewadi | 0.80 | 0.57 |
| *Fejervarya* cf. *brevipalmata* | Chalkewadi | 19.80 | 27.01 |
| *Hoplobatrachus tigerinus* | Dhamapur | 0.80 | 0.28 |
| *Indotyphlus maharashtraensis* | Jagmin | 0.93 | 0.80 |
| *Raorchestes ghatei* | Jagmin | 1.23 | 0.47 |
| *Fejevarya* cf. *sahyadris* | Kudopi | 0.10 | * |
| *Fejevarya* cf. *sahyadris* | Kudopi | 0.10 | * |
| *Gegeneophis* cf. *seshachari* | Kudopi | 0.10 | * |
| *Gegeneophis* cf. *seshachari* | Kudopi | 0.10 | * |
| *Gegeneophis cf. seshachari* | Kudopi | 0.30 | 0.14 |
| *Gegeneophis* cf*. seshachari* | Kudopi | 1.10 | 0.70 |
| *Fejevarya* cf. *sahyadris* | Lanja | 0.30 | 0.14 |
| *Fejevarya* cf*. cepfi* | Lanja | 0.35 | 0.07 |
| *Fejevarya* cf. *sahyadris* | Lanja | 0.10 | 0.00 |
| *Fejevarya* cf. *sahyadris* | Lanja | 0.40 | 0.00 |
| *Fejervarya* sp. | Lanja | 0.30 | * |
| *Indotyphlus* cf*. battersbyi* | Masai | 24.30 | 15.98 |
| *Fejervarya* sp. | Mhavashi | 0.20 | * |
| *Raorchestes ghatei* | Panchgani | 0.65 | 0.12 |
| *Raorchestes ghatei* | Panchgani | 0.90 | 0.57 |
| *Duttaphrynus melanostictus* | Panchgani | 0.90 | * |
| *Sphaerotheca dobsonii* | Ratnagiri | 0.67 | 0.60 |
| *Hoplobatrachus tigerinus* | Shipole | 0.60 | 0.42 |
| *Fejervarya* sp. | Shipole | 0.90 | 0.75 |
| *Hoplobatrachus tigerinus* | Shipole | 0.30 | 0.35 |
| *Hoplobatrachus tigerinus* | Shipole | 0.67 | 0.45 |
| *Hoplobatrachus tigerinus* | Shipole | 0.35 | 0.21 |
| *Indirana* cf. *chiravesi* | Zenda | 2.75 | 2.62 |

**Table 5.** Negative samples after 3 qPCR runs for presence of *Bd*, with species and their plateau locations. NA is the abbreviation for None Accessed.

| **Species** | **Site** | **GE** |
| --- | --- | --- |
| *Duttaphrynus melanostictus* | Amboli Low | NA |
| *Xanthophryne tigerina* | Amboli High | NA |
| *Xanthophryne tigerina* | Amboli High | NA |
| *Xanthophryne tigerina* | Amboli High | NA |
| *Xanthophryne tigerina* | Amboli High | NA |
| *Xanthophryne tigerina* | Amboli High | NA |
| *Xanthophryne tigerina* | Amboli High | NA |
| *Xanthophryne tigerina* | Amboli High | NA |
| *Xanthophryne tigerina* | Amboli Low | NA |
| *Xanthophryne tigerina* | Amboli Low | NA |
| *Xanthophryne tigerina* | Amboli Low | NA |
| *Xanthophryne tigerina* | Amboli Low | NA |
| *Xanthophryne tigerina* | Amboli Low | NA |
| *Xanthophryne tigerina* | Amboli Low | NA |
| *Xanthophryne tigerina* | Amboli Low | NA |
| *Fejervarya* sp. | Amboli Low | NA |
| *Gegeneophis* cf*. ramaswamii* | Amboli Low | NA |
| *Gegeneophis* cf*. ramaswamii* | Amboli Low | NA |
| *Gegeneophis* cf*. ramaswamii* | Amboli Low | NA |
| *Gegeneophis* cf*. ramaswamii* | Amboli Low | NA |
| *Duttaphrynus melanostictus* | Chalkewadi | NA |
| *Fejervarya* cf*. brevipalmata* | Chalkewadi | NA |
| *Fejervarya* cf*. brevipalmata* | Chalkewadi | NA |
| *Fejervarya* sp. | Chalkewadi | NA |
| *Fejervarya* sp. | Chalkewadi | NA |
| *Fejervarya* sp. | Chalkewadi | NA |
| *Fejervarya* sp. | Dhamapur | NA |
| *Fejervarya* sp*.* | Dhamapur | NA |
| *Fejervarya* sp. | Dhamapur | NA |
| *Fejervarya* sp. | Dhamapur | NA |
| *Fejervarya* sp. | Dhamapur | NA |
| *Sphaerotheca dobsonii* | Dhamapur | NA |
| *Sphaerotheca dobsonii* | Dhamapur | NA |
| *Duttaphrynus melanostictus* | Jagmin | NA |
| *Duttaphrynus melanostictus* | Jagmin | NA |
| *Fejervarya* sp*.* | Jagmin | NA |
| *Fejervarya* sp. | Jagmin | NA |
| *Indotyphlus maharashtraensis* | Jagmin | NA |
| *Indotyphlus maharashtraensis* | Jagmin | NA |
| *Raorchestes ghatei* | Jagmin | NA |
| *Microhyla ornata* | Kudopi | NA |
| *Fejervarya* cf. *sahyadris* | Kudopi | NA |
| *Fejervarya* cf. *sahyadris* | Kudopi | NA |
| *Fejervarya* cf. *sahyadris* | Kudopi | NA |
| *Fejervarya* cf. *sahyadris* | Kudopi | NA |
| *Sphaerotheca dobsonii* | Kudopi | NA |
| *Gegeneophis* cf*. seshachari* | Kudopi | NA |
| *Gegeneophis* cf*. seshachari* | Kudopi | NA |
| *Gegeneophis* cf*. seshachari* | Kudopi | NA |
| *Gegeneophis* cf. *seshachari* | Kudopi | NA |
| *Label Damaged* | Label Damaged | NA |
| *Label Damaged* | Label Damaged | NA |
| *Label Damaged* | Label Damaged | NA |
| *Euphlyctis* cf. *cyanophlyctis* | Lanja | NA |
| *Fejevarya* cf*. caperata* | Lanja | NA |
| *Fejervarya* cf*. sahyadris* | Lanja |  |
| *Fejervarya* sp*.* | Lanja | NA |
| *Fejervarya* sp. | Lanja | NA |
| *Hoplobatrachus tigerinus* | Lanja | NA |
| *Hoplobatrachus tigerinus* | Lanja | NA |
| *Gegeneophis seshachari* | Lanja | NA |
| *Hoplobatrachus tigerinus* | Masai | NA |
| *Sphaerotheca dobsonii* | Masai | NA |
| *Indotyphlus* cf*. battersbyi* | Masai | NA |
| *Indotyphlus* cf. *battersbyi* | Masai | NA |
| *Fejervarya* sp. | Mhavashi | NA |
| *Fejervarya* sp. | Mhavashi | NA |
| *Duttaphrynus melanostictus* | Panchgani | NA |
| *Duttaphrynus melanostictus* | Panchgani | NA |
| *Euphlyctis* cf. *cyanophlyctis* | Panchgani | NA |
| *Fejervarya* sp. | Panchgani | NA |
| *Fejervarya* sp. | Panchgani | NA |
| *Fejervarya* sp. | Panchgani | NA |
| *Raorchestes ghatei* | Panchgani | NA |
| *Fejervarya* cf*. sahyadris* | Ratnagiri | NA |
| *Fejervarya* cf. *sahyadris* | Ratnagiri | NA |
| *Unknown* | Ratnagiri | NA |
| *Uperodon globulosus* | Ratnagiri | NA |
| *Fejervarya* cf. *sahyadris* | Shipole | NA |
| *Fejervarya* cf. *sahyadris* | Shipole | NA |
| *Hoplobatrachus tigerinus* | Shipole | NA |
| *Fejervarya* sp*.* | Shipole | NA |
| *Psuedophilautus* sp. | Shipole | NA |
| Unknown | Shipole | NA |
| *Fejervarya* sp. | Zenda | NA |
| *Indirana chiravasi* | Zenda | NA |
| *Indirana chiravasi* | Zenda | NA |
